# V-ATPase-associated prorenin receptor is upregulated in prostate cancer after PTEN loss

**DOI:** 10.18632/oncotarget.27075

**Published:** 2019-08-13

**Authors:** Amro H. Mohammad, Sarah Assadian, Frédéric Couture, Karen J. Lefebvre, Wissal El-Assaad, Veronique Barrès, Veronique Ouellet, Pierre-Luc Boulay, Jieyi Yang, Mathieu Latour, Luc Furic, William Muller, Nahum Sonenberg, Anne-Marie Mes-Masson, Fred Saad, Robert Day, Jose G. Teodoro

**Affiliations:** ^1^ Goodman Cancer Research Center, McGill University, Montréal, Québec, Canada; ^2^ Department of Biochemistry, McGill University, Montréal, Québec, Canada; ^3^ Institut de Pharmacologie de Sherbrooke, Department of Surgery and Urology, Université de Sherbrooke, Sherbrooke, Québec, Canada; ^4^ Centre de Recherche du Centre Hospitalier de l’Université de Montréal (CRCHUM), Institut du Cancer de Montréal, Montréal, Québec, Canada; ^5^ Department of Pathology, CHUM, Université de Montréal, Montréal, Québec, Canada; ^6^ Prostate Cancer Translational Research Laboratory, Peter MacCallum Cancer Centre, Melbourne, Victoria, Australia; ^7^ Cancer Program, Biomedicine Discovery Institute, Department of Anatomy and Developmental Biology, Monash University, Melbourne, Victoria, Australia; ^8^ Sir Peter MacCallum Department of Oncology, University of Melbourne, Melbourne, Victoria, Australia; ^9^ Department of Medicine, Université de Montréal, Montréal, Québec, Canada; ^10^ Department of Surgery, CHUM, Université de Montréal, Montréal, Québec, Canada

**Keywords:** prostate cancer, PTEN, prorenin receptor, soluble prorenin receptor, V-ATPase complex

## Abstract

Phosphatase and tensin homolog (PTEN) tumor suppressor protein loss is common in prostate cancer (PCa). PTEN loss increases PI3K/Akt signaling, which promotes cell growth and survival. To find secreted biomarkers of PTEN loss, a proteomic screen was used to compare secretomes of cells with and without PTEN expression. We showed that PTEN downregulates Prorenin Receptor (PRR) expression and secretion of soluble Prorenin Receptor (sPRR) in PCa cells and in mouse. PRR is an accessory protein required for assembly of the vacuolar ATPase (V-ATPase) complex. V-ATPase is required for lysosomal acidification, amino acid sensing, efficient mechanistic target of Rapamycin complex 1 (mTORC1) activation, and β-Catenin signaling. On PCa tissue microarrays, PRR expression displayed a positive correlation with Akt phosphorylation. Moreover, PRR expression was required for proliferation of PCa cells by maintaining V-ATPase function. Further, we provided evidence for a potential clinical role for PRR expression and sPRR concentration in differentiating low from high Gleason grade PCa. Overall, the current study unveils a mechanism by which PTEN can inhibit tumor growth. Lower levels of PRR result in attenuated V-ATPase activity and reduced PCa cell proliferation.

## INTRODUCTION

The *PTEN* gene is frequently mutated in prostate cancer (PCa) and mutation is even more frequent as cancers progress to metastatic, castration-resistant forms. [1, 2]. Through inhibition of the phosphoinositide 3-kinase (PI3K)/Akt pathway, PTEN can suppress multiple downstream pathways required for tumor growth by preventing activation of the mechanistic target of Rapamycin complex 1 (mTORC1) [3]. mTORC1 is an evolutionary conserved complex that mediates diverse processes including the activation of anabolic metabolism and growth pathways [4]. The mTORC1 complex minimally includes the serine/threonine kinase, mTOR, and an adaptor protein, regulatory-associated protein of mTOR (Raptor). Two of the better-characterized substrates of mTORC1 include ribosomal S6 kinase 1 (S6K1) and the eukaryotic initiation factor 4E binding protein-1 (4EBP1), which enhance cap-dependent translation downstream of PI3K [5, 6].

The PI3K/Akt pathway has been reported to increase lysosomal biogenesis [7, 8]. Among the genes regulated by PI3K pathway are those encoding subunits of the V-ATPase complex [8]. The V-ATPase complex is an essential proton pump required for acidification of lysosomes [9]. In addition to the core V-ATPase subunits, the PI3K pathway also induces expression of the gene encoding the ATPase accessory protein 2 (ATP6ap2) [8]. ATP6ap2, which is also known as the Prorenin Receptor (PRR), is essential for proper V-ATPase complex assembly and function [10].

PRR is a transmembrane protein cleaved by a proprotein convertase to generate two peptides [11]. A secreted N-terminal form, called soluble Prorenin receptor (sPRR), is found in both plasma and urine [12]. A second membrane-associated peptide, designated M8.9, associates with the V-ATPase and is required for proper function of the V-ATPase complex [11]. Due to its critical role in V-ATPase function, PRR is essential for development in mouse, as *ATP6AP2* gene knockout is embryonically lethal [13, 14].

In the current study, using an unbiased proteomic screen, we demonstrate for the first time that PRR is downregulated by PTEN in PCa cells. PRR expression was elevated in human PCa and correlated with phosphorylated Akt staining. PRR expression was required for proper function of V-ATPase and cell proliferation in PCa cells. Of particular interest, we observed that the secreted proteolytic fragment of PRR, the sPRR, is elevated in the urine of men with high Gleason grade PCa. These results demonstrate a hitherto unknown mechanism by which PTEN can downregulate cell growth and highlight a potential novel biomarker to distinguish aggressive and indolent PCa.

## RESULTS

### PTEN regulates multiple prostate cancer cell secreted factors

To discover secreted biomarkers of PTEN mutation and possible cell-non-autonomous effects of PTEN, we performed a screen to compare secretomes of PTEN expressing and non-expressing cells. PTEN expression was reconstituted in PCa LNCaP cells using adenovirus vectors (Supplementary Figure 1A). Proteins from conditioned media (CM) of LNCaP cells infected with adenovirus expressing wild type PTEN (Ad-PTEN WT) or a LacZ control (Ad-LacZ) were identified by 2D-Difference Gel Electrophoresis (2D-DiGE) followed by mass spectrometry (Supplementary Figure 1B and 1C). Fifteen different proteins were identified, 4 upregulated and 11 downregulated in response to PTEN expression (Table 1). Two of the downregulated proteins identified were prostate specific antigen (PSA) and Insulin-like growth factor binding protein 2 (IGFBP-2); which were previously demonstrated to be targets of the PI3K pathway [15, 16], and which validate the screening protocol. From the remaining proteins downregulated by PTEN, the soluble form of the (Pro)renin receptor (sPRR), Spondin 2 (SPON2) and Cystatin C (CST3) were selected for further examination

**Table 1:** List of the PTEN-downregulated and upregulated secreted factors identified in the 2D-DiGE as the number of peptides found in the designated spots on the gel after infecting LNCaP cells with Ad-PTEN WT

Protein name	Abbreviation	Average ratio *PTEN*/*LacZ*	Number of peptides identified	Spots
Prostate specific antigen precursor	PSA	–2.14	17	24–31
(Pro)Renin receptor	PRR	–2.1	5	32
Spondin 2 precursor	SPON2	–1.76	15	12.0–17
Insulin-like growth factor binding protein 2	IGFBP-2	–1.64	17	18, 20–23
Cystatin C	CST3	–1.48	14	33
Alpha-1 collagen VI	COL6A1	–1.46	6	2
Ribonuclease/angiogenin inhibitor 1	RNH1	–1.45	4	10
Putative LAR preprotein	LAR	–1.43	6	3
Calsyntenin 1	CLSTN1	–1.38	27–29	5, 6
Alpha-2-macroglobulin precursor	A2M	–1.35	1	1
Alpha actinin 4	ACTN4	1.37	24	7
Beta-actin	ACTB	1.42	15	11
Desmoglein 2 preprotein	DSG2	1.43	57	8
Chaperonin containing TCP1, subunit 8 (theta)	CCT8	1.52	3	9

### PTEN controls translation and mRNA expression of secreted factors in prostate cancer cells

**Figure 1:**
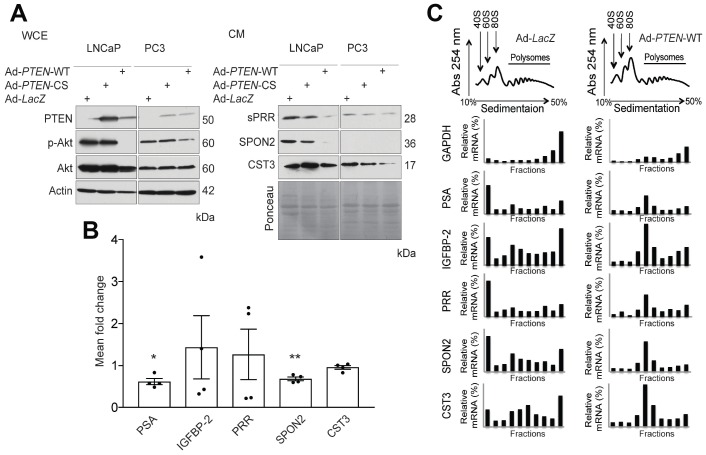
PTEN regulates expression of secreted factors at the translational and transcriptional levels. (**A**) Western Blot analysis demonstrating the effect of functional Phosphatase and Tensin homolog (PTEN) expression on phosphorylated Akt (p-Akt) levels following infection with wild-type PTEN Adenovirus (Ad-*PTEN*-WT), the catalytically inactive PTEN mutant (Ad-*PTEN*-CS), and LacZ (Ad-*LacZ*) control in whole cell extracts (WCE) of LNCaP and PC3 cells. Total Akt was used as a control for p-Akt levels and Actin was used as a loading control (Left Panel). Secretion of soluble Prorenin Receptor (sPRR), Spondin 2 (SPON2) and Cystatin C (CST3) was assessed by western blotting in the conditioned media (CM) of the same cells following infection with the adenoviruses indicated. Ponceau staining was used as the loading control (Right Panel) (*n =* 3). (**B**) qRT-PCR analysis demonstrating mean fold change in the mRNA expression of Prostate Specific Antigen (PSA) (^*^ *P =* 0.014), Inulin-like growth factor binding protein 2 (IGFBP-2), Prorenin Receptor (PRR), SPON2 (^**^ *P =* 0.006), and CST3 in Ad-*PTEN*-WT-infected LNCaP cells normalized to Ad-*LacZ* controls (*n =* 4). (**C**) Polysome profiles of PSA, IGFBP-2, and PRR in Ad-*LacZ* and Ad-*PTEN*-WT-infected LNCaP cells. The levels of transcripts in each fraction were quantified by qRT-PCR. Glyceraldehyde 3-phosphate dehydrogenase (*GAPDH*) was used as a control. Data are presented as the mean ± SEM. Statistical tests conducted using Student’s *t* test.

To validate the putative PTEN-regulated proteins, whole cell extracts (WCE) and CM were collected from LNCaP and PC3 PCa cells infected with Ad-PTEN WT, catalytically inactive mutant PTEN (Ad-PTEN-CS), or Ad-LacZ control (Figure 1A, left panel). sPRR, SPON2 and CST3 were all downregulated in response to WT PTEN expression compared to the inactive PTEN mutant and LacZ controls (Figure1A, right panel). Similar results were observed in PC3 cells although SPON2 was not expressed (Figure 1A, right panel). To determine if the downregulation of the proteins was due to inhibition of translation, polysome profiling was used to assess mRNA translation (Figure 1B). mRNA levels in the fractions was measured by qRT-PCR and revealed a shift of the PSA, IGFBP-2, PRR, SPON2 and CST3 transcripts toward the monosomal population in PTEN-expressing cells compared to the LacZ controls (Figure 1B). This shift was not apparent in a housekeeping gene, Glyceraldehyde 3-phosphate dehydrogenase (*GAPDH*). qRT-PCR was also performed to assess the levels of the mRNAs in Ad-PTEN-WT infected LNCaP cells compared to Ad-LacZ controls. The levels of all transcripts were reduced compared to the infected control cells, however the difference in transcript levels only reached significance for PSA and SPON-2 (Figure 1C). Therefore, PTEN negatively regulates the expression of the secreted factors PSA, IGFBP-2, PRR, SPON2 and CST3 predominantly at the level of mRNA translation.

### PRR expression correlates with phosphorylated Akt levels in prostate cancer

**Figure 2:**
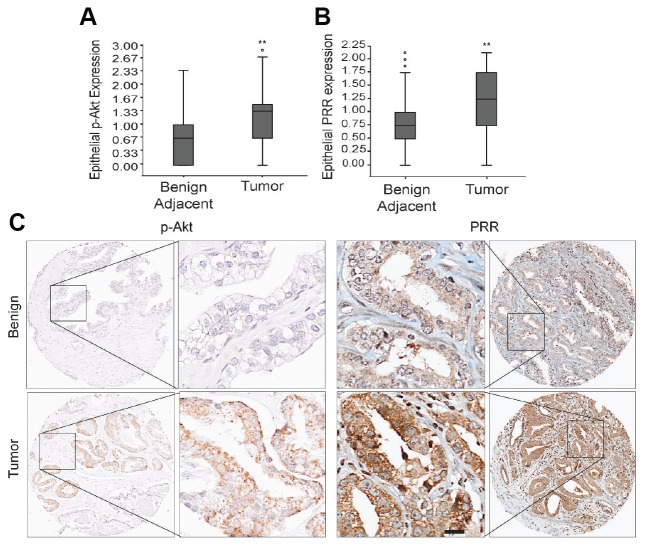
Analysis of p-Akt and PRR expression in prostate cancer tissue microarrays. Box and Whisker plots demonstrating the distribution of (**A**) phosphorylated Akt (p-Akt) (^**^ *P* < 0.01) (benign adjacent, upper extreme (UE)=2.33, upper quartile (UQ)=1.0, median (M)=0.67, lower quartile (LQ)=0.00, lower extreme (LE)=0.00; tumor, outlier (O)=2.82, UE=2.67, UQ=1.45, M=1.33, LQ=0.67, LE=0.00) and (**B**) Prorenin Receptor (PRR) (^**^ *P* < 0.01) (benign adjacent, O=2.1, 1.98, 1.84, UE=1.75, UQ=1.00, M=0.75, LQ=0.50, LE=0.00; tumor, UE=2.15, UQ=1.75, M=1.25, LQ=0.75, LE=0.00) staining intensity scores (0-3) evaluated by IHC in Benign adjacent and tumor tissues from 285 radical prostatectomy specimens arrayed on Tissue microarrays. (**C**) Representative whole and IHC images (20×) of p-Akt and PRR stained benign and tumor tissue microarrays showing the increased expression of PRR and p-Akt in tumor tissue microarrays relative to benign tissue. Scale bar measures 200 μm. Statistical tests conducted using Student’s *t* test.

To validate the identified PTEN-regulated proteins in human PCa, levels of phosphorylated Akt and CST3, PRR, and SPON2 were assessed by IHC on tissue microarrays (TMAs) from a cohort of 285 patients (Supplementary Table 1). Staining intensity was assessed on a scale of 0–3 (Supplementary Figure 2A). The PRR antibody used for IHC was verified to ensure only a unique band was detected (Supplementary Figure 2B and 2C). Of the four PTEN targets identified in our screen, PRR was the only one that correlated positively and significantly with phosphorylated Akt staining (Table 2). Phosphorylated Akt staining was significantly higher in tumor samples compared to adjacent normal tissue (Figure 2A) and positively correlated with several negative clinical outcomes (Supplementary Table 2). Scoring of PRR staining of the TMA showed that PRR expression was significantly higher in tumor core/glands compared to benign adjacent tissue (Figure 2B). This correlation was observed in both benign and tumor cores and was also evident within single cores where PRR upregulation was specific to glands with elevated phosphorylated Akt staining (Figure 2C).

**Table 2:** Correlation values between the PTEN-regulated factors and p-Akt staining in prostate Benign Adjacent (BA) and Tumor (T) tissue

Tissue	Statistical parameter	CST3		PRR		SPON2	
		BA	T	BA	T	BA	T
p-Akt-BA	Correlation Coefficient	–0.024	0.028	0.295^**^	–0.02	–0.143^*^	0.039
	*P* value	0.696	0.647	<0.001	0.736	0.02	0.542
	*N*	266	264	279	276	266	264
p-Akt-T	Correlation Coefficient	0.008	0.077	0.017	0.345^**^	0.074	–0.036
	*P* value	0.895	0.207	0.774	<0.001	0.234	0.559
	*N*	264	267	272	277	263	267

### PTEN downregulates PRR expression and sPRR secretion

**Figure 3:**
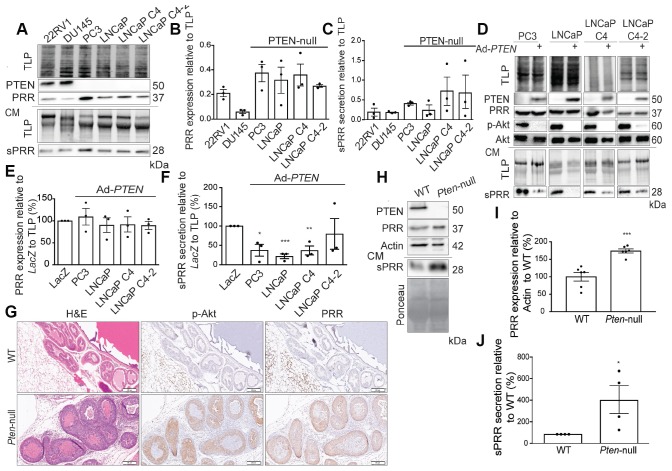
PTEN downregulates PRR expression and sPRR secretion. (**A**) Western blot analysis showing more endogenous Prorenin Receptor (PRR) expression in whole cell extracts and soluble Prorenin Receptor (sPRR) secretion in conditioned media (CM) of Phosphatase and tensin homolog (PTEN)-null prostate cancer lines (PC3, LNCaP, LNCaP C4, and LNCaP C4-2) than in PTEN-expressing lines (22RV1 and DU145). Total lane protein (TLP) was used as a loading control. Dot plots of the quantification of (**B**) PRR expression and (**C**) sPRR secretion relative to total lane protein in the same prostate cancer lines. (**D**) Western blot analysis showing that PTEN reintroduction in PTEN-null lines via PTEN adenovirus infection (Ad-*PTEN*) reduces sPRR secretion when compared to their *LacZ*-infected counterparts. PTEN reintroduction also reduces Akt phosphorylation as expected. Total Akt was used as a control for phosphorylation of Akt (p-Akt) and TLP was used as a loading control. (**E**) Dot plots of the quantification of PRR expression in the Ad-*PTEN*-WT-infected prostate cancer lines relative to the *LacZ*-infected counterparts and to TLP, demonstrating largely unchanged levels of PRR expression after Ad-*PTEN* infection. (**F**) Dot plots of the quantification of sPRR secretion shows significant reductions in the Ad-*PTEN*-WT-infected PC3 (^*^ *P =* 0.015), LNCaP (^***^ *P =* 0.0002), LNCaP C4 (^**^ *P =* 0.005), and LNCaP C4-2 relative to the *LacZ*-infected counterparts and to TLP (*n =* 3). (**G**) Representative IHC images of Wild type (WT) and *Pten*-null (*Pten*^-/-^) mouse prostate sections after H&E staining, and p-Akt and PRR IHC staining. (**H**) Western blot analysis demonstrating increased PRR expression in whole cell extracts, and sPRR secretion in CM of *Pten*^-/-^ mouse embryonic fibroblasts compared to wild type (WT) fibroblasts. Actin was used as a loading control in whole cell extracts and Ponceau was used as loading control for CM. Dot plots of the quantification of (**I**) PRR expression in *Pten*^-/-^ (^***^ *P =* 0.0001, *n =* 6) and of (**J**) sPRR secretion in *Pten*^-/-^ (^*^ *P =* 0.025, *n =* 4) in mouse embryonic fibroblasts relative to wild type counterparts. Scale bar measures 200 μm. Data are presented as the mean ± SEM. Statistical tests conducted using Student’s *t* test.

PRR is a 350-amino acid transmembrane protein that is cleaved in the trans-Golgi by Furin to yield an 8.9 kDa fragment (called M8.9), and a secreted soluble fragment, sPRR [17]. To further characterize the relationship between PTEN and expression of sPRR and PRR, we analyzed expression in six PCa cell lines. The 22RV1 and DU145 cell lines express PTEN, whereas PC3, LNCaP, and the LNCaP derivatives are PTEN-null. The cell lines expressing PTEN (22RV1 and DU145 lines) have less endogenous levels of PRR and less sPRR secretion. Conversely, PTEN-null cell lines (PC3 and LNCaP lines) express more endogenous PRR and secrete more sPRR (Figure 3A–3C). Reconstitution of PTEN using adenoviral infection resulted in reduced PRR expression in LNCaP C4 cells but reduced sPRR secretion in all lines (Figure 3D–3F). Therefore, PTEN may control sPRR secretion through regulating expression of PRR or by directly affecting the processing of PRR into sPRR.

PRR expression was also examined in mice with prostate specific deletion of PTEN [18]. As expected, PTEN deletion resulted in higher levels of phosphorylated Akt staining in the prostates (Figure 3G). PRR was also expressed at a much higher level in *PTEN*^–/–^ mice compared to the WT controls (Figure 3G). Similarly, mouse embryonic fibroblasts from PTEN-null animals showed elevated PRR expression and sPRR secretion compared to wild type cells (Figure 3H–3J).

### Downregulation of PRR results in a decrease in cell growth and V-ATPase activity

**Figure 4:**
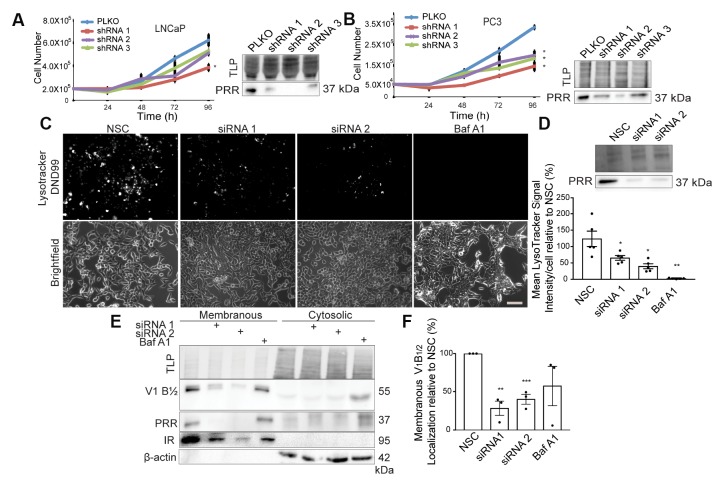
Downregulation of PRR results in a decrease in cell growth and V-ATPase activity. (**A**) Growth curves and Western blot analysis of LNCaP cells infected with empty vector (PLKO), Prorenin Receptor (PRR) shRNA 1 (^*^ *P =* 0.034), shRNA 2, or shRNA 3 (Left Panel) (*n =* 3). (**B**) Growth curves and Western blot analysis of PC3 cells infected with PLKO, PRR shRNA 1 (^*^ *P =* 0.017), shRNA 2 (^*^ *P =* 0.042), or shRNA 3 (^*^ *P =* 0.021) (Left Panel) (*n =* 3). Total lane protein (TLP) was used as the loading control. (**C**) LysoTracker DND-99 and corresponding brightfield images (20×) of NSC, PRR siRNA1, or siRNA 2-transfected, and Bafilomycin A1(Baf A1)-treated LNCaP C4-2 cells. (**D**) Quantification of Mean LysoTracker signal per cell showing a reduction in LysoTracker signal after transfection with PRR siRNA 1 (^*^ *P =* 0.037), siRNA 2 (^*^ *P =* 0.048), or Baf A1 (100 nM) (^**^ *P =* 0.006) treatment in LNCaP C4-2 cells (*n =* 5). Chemical inhibition with Baf A1 serves as a positive control. Western blot analysis validating PRR knockdown in siRNA1 and siRNA2-transfected LNCaP C4-2 cells. TLP was used as a control (Top Panel). (**E**) Western blot analysis showing less expression of the V-ATPase B1/2 subunits and less localization in the membranous fraction of LNCaP cells following PRR knockdown. Chemical inhibition with Baf A1 (100 nM) increases cytosolic availability of the V1 domain, which is indicative of less V-ATPase assembly at the membranous fraction. Insulin Receptor (IR) and Actin localization were used to validate fractionation. TLP was used as a loading control. (**F**) Corresponding quantification of V-ATPase B1/2 membranous localization in NSC, PRR siRNA 1 (^**^ *P =* 0.0015), siRNA 2 (^***^ *P =* 0.0008), or in Baf A1 (100 nM) treated cells (*n =* 3). Scale bar measures 100 μm. Data are presented as the mean ± SEM. Statistical tests conducted using Student’s *t* test.

To determine if lower PRR expression affects the growth of PCa cells, PRR was knocked down using three different shRNAs and cell proliferation was measured. PRR knockdown resulted in decreased cell growth in both LNCaP and PC3 cells (Figure 4A and 4B). PRR is believed to function as an essential accessory factor for the V-ATPase complex, which has been functionally linked to cell proliferation [19]. We therefore determined if PRR knockdown would result in decreased V-ATPase activity in PCa cells. PRR was knocked down in LNCaP cells and vacuolar acidification was quantitated based on the uptake of *LysoTracker Red DND-99* probe. Quantification of Lysotracker signal showed significantly reduced acidification in PRR-knockdown cells compared to non-silencing controls (Figure 4C and 4D). Similar results were observed using HEK 293T cells upon PRR knockdown (Supplementary Figure 3A and 3B). Conversely, overexpression of PRR significantly increased the uptake of the LysoTracker probe (Supplementary Figure 3C and 3D).

To understand how PRR promotes V-ATPase activity, we examined V-ATPase assembly by analyzing the localization of the V_1_ B_1/2_ subunits of the V-ATPase complex. The cytosolic V_1_ domain of the V-ATPase complex uses ATP hydrolysis to power the membrane localized V_0_ domain, which translocates protons into the lumen. V-ATPase assembly was assessed following PRR knockdown by separating the soluble and membranous fractions and immunoblotting for the V_1_ B_1/2_ subunit. Insulin receptor (IR) and β-actin were used to validate separation of both membranous and cytoplasmic fractions, respectively. PRR knockdown resulted in decreased membranous and cytosolic localization of the V_1_ domain suggesting less overall V-ATPase integrity (Figure 4E and 4F). Chemical inhibition of the V-ATPase complex using Bafilomycin A1 inhibited V-ATPase assembly but not subunit expression, as V_1_ B_1/2_ localization was decreased in membranous fraction but increased in the cytosolic fraction. These results are consistent with previous reports suggesting that PRR acts as a chaperone for the V-ATPase required for integrity of the complex [10]. Taken together, these observations show that regulation of PRR expression can directly affect V-ATPase activity.

### PRR expression increases in tumor tissue, correlates with Gleason score, and sPRR is elevated in urine of patients with aggressive prostate cancer

**Figure 5:**
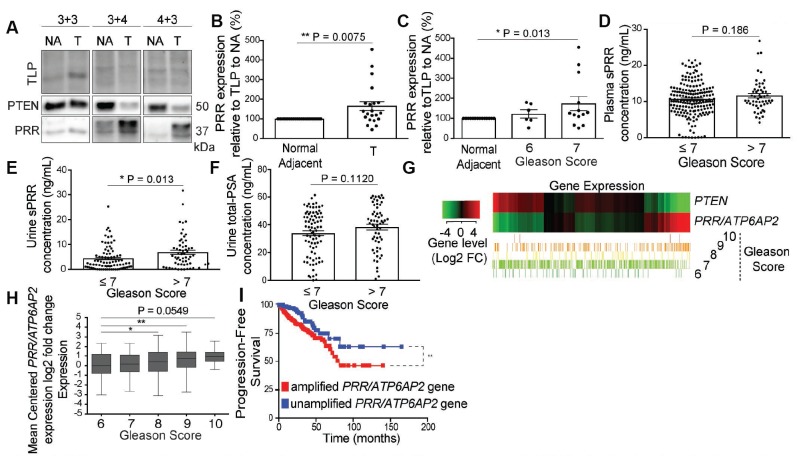
PRR expression increases in tumor tissue, correlates with Gleason score, and sPRR is elevated in urine of patients with aggressive prostate cancer. (**A**) Representative Western blot analysis demonstrating dampened Phosphatase and tensin homolog (PTEN) expression in tumor (T) tissue of patient prostates with Gleason grade 3+4 and 4+3 but not in 3+3 Gleason grade compared to normal adjacent tissue (NA) from the same prostate of each patient. Also, Prorenin Receptor (PRR) expression is increased in the tumor tissue in which PTEN expression is decreased (3+4, and 4+3 Gleason grade patients). Total lane Protein (TLP) was used as a loading control. (**B**) Quantification of PRR expression shows an increase in PRR expression in the tumor tissue compared to normal adjacent across all the Gleason grades (^**^ *P =* 0.0075) (*n =* 21). (**C**) Stratification of PRR expression over Gleason grades 6 (*n =* 6) and 7 (*n =* 13) from the same patients shows a significant increase in PRR expression in Gleason 7 (^*^ *P =* 0.042) but not in Gleason 6 patients. (**D**) ELISA on Plasma samples from prostate cancer patients shows no significant difference in soluble Prorenin Receptor (sPRR) concentration (ng/mL) between Gleason grade groups ≤7 (*n =* 190) and >7 (*n =* 53) (*P =* 0.186). ELISA analysis shows a significant increase in sPRR concentration (^*^ *P =* 0.013) (**E**) but not in prostate specific antigen (PSA) concentration (*P =* 0.112) (**F**) (ng/mL) in urine samples from Gleason score >7 (*n =* 60) compared to ≤7 (*n =* 83) patients. Data are presented as the mean ± SEM. Statistical tests conducted using Student’s *t* test. (**G**) Heat map showing the mRNA expression profile of *PTEN* and *PRR/ATP6AP2* genes across 551 genomic data commons (GDC) of the cancer genome atlas (TCGA) prostate adenocarcinoma (PRAD) tumors, classified based on unsupervised hierarchical clustering into Gleason scores 6 (*n =* 51), 7 (*n =* 287), 8 (*n =* 67), 9 (*n =* 142), and 10 (*n =* 4). (**H**) Box and Whisker plots showing the log 2-fold differential expression of the gene *PRR/ATP6AP2* across TCGA samples grouped by 6 (upper extreme = 2.30, upper quartile = 1.16, median *=* 0.04, lower quartile = –0.90, lower extreme = –3.09), 7 (2.26, 1.07, 0.07, –0.70, –2.71), 8 (3.07, 1.31, 0.32, –0.65, 3.15) (^*^ *P =* 0.02), 9 (3.43, 1.38, 0.70, –0.29, –2.81) (^**^ *P =* 0.002), and 10 (2.51, 1.29, 0.88, 0.36, –0.51) (*P =* 0.054) Gleason scores. Significance was determined by the Wilcoxon rank *T*-Test as compared to Gleason-6. (**I**) Kaplan-Meier plot showing less progression-free survival of TCGA prostate adenocarcinoma patients with an amplified *PRR/ATP6AP2* gene set (*n =* 321) compared to patients with an unamplified *PRR/ATP6AP2* gene set (*n =* 170) (^**^ *P* < 0.0086). Statistical analysis was conducted using the Logrank test to compare the survival distributions of the two patient populations.

To determine the clinical significance of PRR and sPRR in PCa and assess their utility as biomarkers, we analyzed expression of the proteins in patient biosamples. Tumor and normal adjacent tissue from 20 PCa patients (Supplementary Table 3) were homogenized and analyzed for PRR expression. PRR expression was higher in tumor tissue in which PTEN expression was lost, compared to normal adjacent tissues (Figure 5A and 5B). PRR expression also increased with Gleason grade across the samples tested (Figure 5C). To determine if sPRR is a biomarker for aggressive PCa, we quantitated sPRR in the plasma from 243 patients (Supplementary Tables 1 and 4) using an ELISA. Although sPRR concentrations were slightly elevated with higher Gleason grade, the effect was just below statistical significance (Figure 5D). However, analysis of sPRR levels in urine revealed a significant increase in sPRR concentration in the high Gleason grade cohort (Figure 5E). PSA levels in the urine of the same cohort did not show a significant change in PSA concentration (Figure 5F). Since PTEN negatively regulated the mRNA of PRR (Figure 1C), we analyzed the expression of PRR and PTEN in the cancer genome atlas (TCGA)-prostate adenocarcinoma (PRAD) dataset and found that PRR mRNA levels negatively correlate with PTEN expression in most tumors analyzed and to a great extent in high-Gleason score tumors (Figure 5G). Taken individually, PRR mRNA levels also increased with Gleason score (Figure 5H). Further, according to TCGA data acquired (explained in [21]), *PRR/ATP6AP2* inactivating mutations are extremely rare in different malignancies [21], pointing at a critical role for PRR in tumor cells. Interestingly, although the alteration frequency of the *PRR/ATP6AP2* gene is low in PCa, the vast majority of these alterations consist of gene amplifications (TCGA-PRAD, Cell 2015). TCGA analysis of PCa patients with amplification in the *PRR/ATP6AP2* gene shows less progression-free survival than in patients with no alterations in the *PRR/ATP6AP2* gene (Figure 5I). Together these findings suggest that PRR expression correlates with PCa severity and that PRR-related mechanisms may contribute to disease progression.

## DISCUSSION

In the current study, we identified several secreted factors regulated by PTEN. Among the factors identified, PRR expression was found to correlate positively with phosphorylated Akt staining in PCa tissue. We demonstrated for the first time that PRR levels are elevated in PCa and can regulate proliferation of PCa cells. The effect of PRR expression on cell growth is likely due to PRR association with the V-ATPase complex and in support of this model, we showed that PRR could modulate V-ATPase activity in PCa cells.

In addition to sPRR, our screen identified other factors that have implications in PCa. We show that PTEN controls the secretion of Prostate Specific Antigen (PSA) and IGFBP-2 (Table 1). Both of these proteins have been previously reported to be regulated by PTEN and nicely validate the screen [15, 16]. The screen also identified SPON2 as a PTEN-regulated ffactor secreted by PCa cells (Table 1, Figure 1A, right panel). Interestingly, another independent study reported that SPON2 may be an effective serum marker for PCa, when compared to PSA [22].

Measurement of sPRR levels in the plasma of low Gleason grade patients compared to high displayed no statistically significant changes (Figure 5D). However, sPRR was significantly elevated in the urine of high Gleason grade patients (Figure 5E). A possible explanation for the difference in plasma and urine sPRR is that PRR is ubiquitously expressed and, unlike PSA, is not prostate-specific. Any elevation of sPRR levels contributed by PCa are likely masked by the already-high levels of circulating sPRR, whereas sPRR leaking into the urine can readily be detected over background.

There is a great clinical need to identify patients most at risk of developing aggressive forms of PCa. About one third of the men who have elevated PSA levels (>10 ng/mL) have no evidence of PCa at biopsy [23]. Similarly, in approximately 60% of PCa patients’ serum levels of PSA remains low (<3 ng/mL) [24]. PSA screening can lead to unnecessary invasive biopsies and in some cases is unable to detect advanced cancer. Early PCa screening requires more effective biomarkers that faithfully distinguish benign PCa from life-threatening metastatic progression. Elevated sPRR levels in PCa patients’ urine samples could potentially complement PSA measurement in this regard and provide a better understanding of the nature of the disease.

Mutations in the PI3K pathway are common in PCa. Approximately 30% of castration resistant prostate cancers (CRPCs) carry mutations in PI3K [25]. Similarly, homozygous deletions of the *PTEN* gene are found in 13% of localized PCa and in up to 39% of metastases [26–30]. Currently, there are no convenient biomarkers to identify patients who bear tumours with hyperactive PI3K signalling other than IHC analysis of needle biopsies. Factors such as sPRR, could serve as biomarkers that could be used to identify men who carry PTEN and PI3K mutations and are at greater risk to metastatic progression.

As its name implies, PRR is a putative receptor required for processing of Pro-Renin to Renin, which converts Angiotensinogen to Angiotensin to regulate blood pressure [31]. However, PRR appears to have functions beyond the Renin Angiotensin system since knockout of the gene in mouse results in early embryonic lethality [13, 14]. PRR is critical for the assembly and functioning of the V-ATPase complex [10]. Activity of the V-ATPase is required to acidify intracellular vacuoles, such as lysosomes, and maintenance of the pH gradient across the luminal/cytoplasmic junction [32].

In the presence of amino acids, the V-ATPase complex is required for recruitment of mTORC1 to the lysosomal surface, making mTORC1 available for activation by the PI3K/Akt downstream target, Rheb [33]. By downregulating PRR expression, PTEN can control the V-ATPase-mediated mechanism of amino acid sensing and mTORC1 activation. The effects of V-ATPase in cellular signaling likely go far beyond mTORC1 signaling. A recent study identified PRR as a factor required for β-Catenin signaling [34]. Targeting PRR was sufficient to inhibit β-Catenin signaling and to interrupt *Xenopus* development [34]. Thus, PRR may serve a role in integrating multiple growth-promoting pathways. Downregulation of PRR expression by PTEN, may therefore be a general mechanism to suppress cell growth mediated by multiple pathways.

## MATERIALS AND METHODS

### Cells, viruses and western blots

LNCaP cells (ATCC #: CRL-1740) were grown in RPMI 1640 1x supplemented with 15% fetal bovine serum (FBS) and Gentamycin (Wisent). PC3 cells (ATCC#: CRL-1435) were grown in RPMI 1640 (Wisent) supplemented with 10% FBS and Gentamycin. HEK-293T cells (ATCC #: CRL-3216) were grown in DMEM media supplemented with 10% FBS and Gentamycin. Adenovirus expressing wild-type PTEN, mutant PTEN (C124S), or b-galactosidase (LacZ) have been previously described [35, 36]. To obtain conditioned media (CM) following infection, cells were incubated for 24 h and were then washed in PBS and subsequently incubated in fresh RPMI containing 1% insulin transferrin and selenium (ITS, Wisent). CM was then collected after 24 h of conditioning. Whole cell extracts (WCE), obtained by harvesting cells, were boiled in 1X Laemmli buffer. Equal amounts of protein from WCE and CM were resolved by SDS-PAGE. Immunoblots were performed using the following antibodies: PTEN (#9559, Cell Signaling, Phospho-Akt (#9271, Cell Signaling), Total Akt (#4691, Cell signaling), Actin (A2228, Sigma), Cystatin C (AF1196, R&D systems), Spondin2 (AV52336, Sigma), and PRR (GTX114169, GeneTex). Where indicated, western blots used *TGX Stain-Free* (Biorad) imaging of total lane protein (TLP) as loading control according to manufactures protocols.

### Secretome analysis by 2D-difference gel electrophoresis

cM-precipitated proteins were labelled with CyDye DiGE Fluor minimal dyes following the manufacturer’s protocol (GE Healthcare, UK). Electrophoresis was performed using the Ettan DALTsix electrophoresis unit (GE Healthcare). Gels were scanned using the Typhoon Trio+ scanner (GE Healthcare) and analyzed using DeCyder image analysis software (GE Healthcare). The following criteria were used to identify spots of interest: 1) spot must appear on at least 3 of 4 gels, 2) average ratio of protein abundance of PTEN over LacZ must be greater than 1.3 or less than –1.3, and 3) the difference in protein abundance must be statistically significant over four replicates. Spots matching these criteria were then cut and sent for mass spectrometry analysis. See Supplementary Materials and Methods for additional details.

### Conditioned media protein precipitation procedure

for 2D-DiGE experiments, 3 volumes of Ethanol were mixed with the CM and centrifuged for 40 minutes at 4750 rpm at 4° C. The resulting pellet was washed with methanol, left to air dry, and was resuspended overnight in DiGE lysis buffer (7M Urea, 2M Thiourea, 4% CHAPS, 30 mM Tris, 5 mM DTT, pH 8.5) on a thermal-shaker at 22° C. For Western Blot validation of the secreted factors identified by 2D-DiGE and Mass Spectrometry, WCE and CM were collected as described above. Proteins were precipitated from CM by adding trichloroacetic acid (TCA) at a final concentration of 15%. Samples were incubated for 2 h on ice and centrifuged at 10,000 g for 10 minutes. Protein pellets were washed twice with tetrahydrofuran, air dried, and dissolved in resuspension buffer (7M Urea, 2M Thiourea, 4% CHAPS, 5mM Tris pH 8). Protein concentration in CM was quantified using the 2D-Quant Kit (Roche). Equal amounts of protein from WCE and CM were resolved by SDS-PAGE.

### Quantitative real-time PCR

Total RNA was isolated from experimental samples using TRIZOL (Invitrogen) according to the manufacturer’s protocol (Invitrogen, CA, USA). Reverse transcription was performed using the Quantitect Reverse Transcription kit (Qiagen). PCR reactions were carried out with the Quantitect SYBR Green PCR kit (Qiagen) in a Realplex^2^ instrument (Eppendorf). The 18S ribosomal RNA was used as an internal control. Fold induction was calculated relative to samples infected with the control LacZ adenovirus.

### Polysome profiling

For polysome profiling, LNCaP cells infected with Ad-PTEN or Ad-LacZ were treated with 100 μg/mL cycloheximide (CHX) 48 hours post infection. Polysomes were isolated as previously described [37]. RNA was isolated from the polysome fractions using TRIzol (Invitrogen) and quantitative RT-PCR was performed as described above using the primers detailed in Supplementary Table 5. The relative amount of transcript in each fraction was normalized to the Ct values of the first LacZ fraction.

### Prostate-specific PTEN-knockout mouse model, IHC of mouse prostates, and mouse embryonic fibroblasts

The generation and validation of the *Pten*^loxp/loxp^ mouse model is previously described [38]. *Pten*^loxp/loxp^ mice were crossed with male probasin-Cre mice (PB-Cre4) (PB-Cre4 mice described in [39]) for prostate-specific *Pten* gene targeting [40]. Paraffin-embedded urogenital systems isolated from 2-month old WT (*Pten*^loxp/loxp^) mice or from mice with prostate-specific PTEN deletion (*Pten*^loxp/loxp^; PB-Cre4) were generated as previously described [18]. See Supplementary Materials and Methods for mouse section immunohistochemistry procedure details. MEFs derived from *Pten*^loxp/loxp^ mice were infected with adenovirus type 5-Cre (Microbix Biosystems Inc., Toronto, Canada) for postnatal PTEN deletion (*Pten*^loxp/loxp^; PB-Cre4^+^) or adenovirus type 5-GFP as control (Greber and Hemmi laboratories, Zurich, Germany) (*Pten*^loxp/loxp^; PB-Cre4^−^). Infections were carried out at a multiplicity of infection of 50 [40].

### Tissue microarrays (TMAs)

Prostate specimens from radical prostatectomy were obtained from 285 patients operated between 1993 and 2006 at the Centre Hospitalier de l’Université de Montréal (CHUM) (Supplemental Table 1). TMA TF123 was constructed at the Centre de recherche du CHUM (CRCHUM) using specimens from patients included in the CRCHUM-RRCancer prostate cancer biobank. To create the TMA, a pathologist reviewed a hematoxylin/Eosin (H&E)-stained slides from an archived formalin fixed paraffin embedded (FFPE) tissue block and circled the representative tumor area. Two cores of 0.6 mm from each specimen, one selected from the tumor area (T) and the second one from the adjacent non-malignant tissue (BA) were arrayed on a receiver TMA block containing 100 patients. Each TMA was created in duplicate using the TMArrayer (Pathology Devices, Inc., Westminster, MD, USA). The clinico-pathological characteristics of these patients can be found in Supplementary Table 1. See Supplementary Materials and Methods for details on TMA immunohistochemistry.

### shRNA-mediated knockdown of PRR and proliferation

shRNA vectors (Supplementary Table 6) or SHC002 MISSION Non-Target shRNA control vector (Sigma) along with lentivirus packaging plasmids pCMV-dR8.2 and pCMV.VSV.G (Addgene) were transfected in HEK293T/17 (ATCC #CRL: 11268). Target LNCaP, PC3, or RWPE-1 cells (ATCC #: CRL-11609) grown in Keratinocyte-SFM 1X (Gibco) were seeded 24 hours following transfection. Supernatants from HEK293T/17 cells were collected 48 hours after transfection, passed through a 0.45-μm SFCA filter, and applied on target cells along with Polybrene (8 μg/mL) (Sigma). Cells were selected with puromycin (Wisent) for 72 hours. Immediately following selection, cells were plated for growth assays and counted at the times indicated.

### Lysotracker assay and V-ATPase assembly

LNCaP C4-2 and HEK293T cells were transfected with the PRR and control siRNAs. Briefly, 48 hours following Lipofectamine transfection with 50 nM of PRR siRNA (Supplementary Table 6) or Non-silencing control (Sigma), cells were incubated with *LysoTracker (DND-99)* Red (100 nM) (Molecular Probes, Invitrogen) for 50 minutes. Where indicated, cells were treated with 100 nM Bafilomycin (Invitrogen) for 1 hour before the addition of *LysoTracker*. See Supplementary Materials and Methods for details on LysoTracker image processing.

To examine V-ATPase assembly, LNCaP C4-2 cells were homogenized in NP-40 lysis buffer (0.5% NP-40, 50 mM Tris-Hydrochloric acid, 150 mM Sodium Chloride) supplemented with 50 mM Sodium Fluoride, 10 mM Glycerol-2-Phosphate, 100 μM Sodium Orthovanadate, and 1 Roche Protease Inhibitor mix tablet (Roche) per 10 ml lysis buffer. Lysate was centrifuged at 500 g for 10 minutes at 4° C. Supernatant was then centrifuged at 100,000 g for 30 minutes at 4° C. Pellets containing membrane fractions were homogenized again with the same buffer as above. The supernatant containing the cytosolic fraction was concentrated using Amicon Ultra filter with 10-kDa cut off (Millipore). For immunoblots, antibodies for V-ATPase 1 B1/2 subunits (sc-55544, Santa Cruz) and Insulin Receptor (L55B10, Cell Signaling) were used.

### Human prostate tumor lysates

Prostate tissues used for immunoblots were freshly dissected (within 30 minutes) from prostate specimens after radical prostatectomies performed at the Centre Hospitalier Universitaire de Sherbrooke. The clinico-pathological parameters for these patients can be found in Supplementary Table 3. See Supplementary Materials and Methods for details on tumor tissue sample preparation.

### TCGA prostate cancer mRNA levels

Bioinformatic analyses of the TCGA PCa Adenocarcinoma (Provisional study 2015) were performed on TCGA tumor samples (*n =* 548) using both RNA-sequencing and clinical features. Briefly, level 3 log2(x + 1) transformed RSEM normalized gene expression RNASeqV2 and clinical annotations were downloaded from cBioPortal (http://www.cbioportal.org) [20, 41]. See Supplementary Materials and Methods for information on how clinical relevance with *PRR/ATP6AP2* gene was assessed.

Bioinformatic analyses of the genomic data commons (GDC) of TCGA-PRAD database were performed on tumor samples. Briefly, log2(FPKM-UQ+1)^1^ RNA_Seq normalized data, tumor sample identifiers, clinical Gleason Score annotations, and GDC TCGA-PRAD *PTEN* and *PRR/ATP6AP2* mRNA levels were downloaded from UCSC Xena hubs https://gdc.xenahubs.net. mRNA expression analyses were performed on tumor samples using the clinical Gleason Score annotation. Unsupervised hierarchical clustering analysis was based on the average linkage and Euclidean distances as the similarity metric. The R console (version R 3.3.2), Bioconductor source (http://bioconductor.org) was used to perform microarray data analysis [42–46].

### Quantitating sPRR and Total-PSA concentration

An ELISA assay (IBL, International) was used to measure concentrations of sPRR in plasma and urine samples from PCa patients according to manufacturer’s protocol (IBL International, Japan). Total PSA was also measured using an ELISA kit (Sigma, RAB0331) according to the manufacturer’s protocol.

### Statistical analysis

To evaluate the correlation between the different parameters, a non-parametric Spearman correlation test was used. For heatmap analysis, significance was determined by Linear Models for Microarray Analysis^5^ cutoff setting *P* value < 0.01. For other statistical tests, the two-tailed Student *t*-test was used to test significance. *P*-values below 0.05 were considered statistically significant.

### Ethics statement

All patients signed an informed consent form and the Centre de Recherche du Centre Hospitalier de l’Universite de Montreal ethics committee (CE 15.366) as well as the Institutional Review Committee for the use of human resected material at the Centre Hospitalier Universitaire de Sherbrooke (approval #10-017) approved the use of these patient specimens for biomarker evaluation**.**

## SUPPLEMENTARY MATERIALS



## Abbreviations

ACTN4: Alpha actinin 4
ATP6AP2: ATPase-associated protein 2
CD: cytoplasmic domain
CLSTN1: Calsyntenin 1
CM: conditioned media
COL6A1: Alpha-1 collagen VI
CST3: Cystatin C
CRPC: Castration resistant prostate cancer
ELISA: enzyme-linked immunosorbent assay
FFPE: formalin-fixed and paraffin-embedded
GAPDH: Glyceraldehyde 3-phosphate dehydrogenase
GDC: Genomic data commons
HEK293: Human embryonic kidney 293
MEFs: mouse embryonic fibroblasts
mTORC1: mechanistic target of Rapamycin complex 1
P70S6K: P70S6 kinase
PCa: prostate cancer
PI3K: phosphoinositide-3 kinase
PIP_2_: phosphatidylinositol (4,5)-biphosphate
PIP_3_: phosphatidylinositol (3,4,5)-triphosphate
PRAD: Prostate adenocarcinoma
PRR: Prorenin Receptor
PSA: prostate specific antigen
PTEN: Phosphatase and Tensin Homolog
Raptor: regulatory protein associated with mTOR
Rheb: Ras homolog enriched in brain
SPON2: Spondin2
sPRR: soluble Prorenin Receptor
TCGA: the cancer genome atlas
TLP: total lane protein
TM: transmembrane
TMA: tissue microarray
V-ATPase: vacuolar-ATPase
WCE: whole cell extract
2D-DiGE: 2D-Difference Gel Electrophoresis
4EBP1: eukaryotic translation initiation factor 4E-binding protein 1
