# Supplementary material for: V-ATPase-associated prorenin receptor is upregulated in prostate cancer after PTEN loss
Source: Oncotarget. 2019 Aug 13;10(48):4923–36. doi: 10.18632/oncotarget.27075 (PMC6697641; doi:10.18632/oncotarget.27075)
Supplement: Supplementary file 1 [file oncotarget-10-4923-s001.pdf]

V-ATPase-associated prorenin receptor is upregulated in prostate cancer after PTEN loss

SUPPLEMENTARY MATERIALS

2D-difference gel electrophoresis (DiGE)

A pooled internal standard, consisting of 25 µg of protein of each replicate was labelled with Cy2. Experimental samples were combined along with the pooled internal standard, 4.5 µL of 1% immobilized pH gradient (IPG) buffer (GE Healthcare), 5.4 µL of DeStreak Reagent (GE Healthcare), and rehydration buffer (7M Urea, 2M Thiourea, 4% CHAPS, 0.002% Bromophenol blue). Samples were then loaded onto 24 cm pH 3-11 non-linear Immobiline DryStrips (GE Healthcare). Active rehydration and isoelectric focusing (IEF) were performed using the Ettan IPGphor IEF system (GE Healthcare). Immediately following IEF, the IPG strips were equilibrated in equilibration buffer (100 mM Tris, 6M Urea, 30% Glycerol, 2% SDS, 0.002% Bromophenol blue), first in buffer containing 5 mg/mL DTT, then in equilibration containing 45mg/mL iodoacetamide. IPG strips were loaded onto 12.5% SDS-PAGE gels and sealed in place using 1% agarose.

Tissue microarrays (TMAs) immunohistochemistry

Tissue sections of 4µm from TMAs were deparaffinized, rehydrated and incubated in a methanol/0.3% H2O2 solution. Immunostaining for phosphorylated Akt was performed as in mice tissues (below). Immunostaining for PRR, SPON-2, and CST3 was performed using a XT autostainer (Ventana Medical System Inc.). Antigen retrieval was obtained using Cell Conditioning 1 (Ventana Medical System Inc.) for 60min. Antibody dilutions (PRR: Genetex, 1: 100; SPON-2: R&D System, 1:500; CST3: R&D System, 1:500) were manually added to the slides and incubated at 37° C for 60 minutes. Reactions for PRR were developed using the UltraView universal DAB detection kit (Ventana Medical System Inc.). For SPON-2 and CST3 IHCs, however, pre-diluted anti-goat secondary antibody (Santa-Cruz) was automatically dispensed and reactions were developed using the iView DAB detection kit without secondary antibody (Ventana Medical System Inc.). Counterstaining was performed with Hematoxylin and bluing reagent (Ventana Medical System Inc.). All sections were scanned using a VS-110 microscope with a 20× 0,75NA objective and a resolution of 0,3225 µm (Olympus). Images were analyzed with the OlyVIA software (Olympus). The intensity of staining in epithelial cells was assessed based on the following scale: negative or very weak (0), weak (1+), intermediate (2+) and elevated (3+). Final values for each core were calculated as the average of the scores determined by two independent blinded scorers.

Immunohistochemistry (IHC) of mouse prostates

Four-micrometer sections were used for H&E staining and IHC. For IHC, slides were heated for 20 minutes at 60˚C, deparaffinized in xylenes and rehydrated in ethanol gradient. Antigen retrieval was performed using sodium citrate pH 6.0 (p-AKT) or EDTA pH 8.0 (PRR) buffer in a pressure cooker (Cuisinart). Endogenous peroxidase was deactivated with 3% H_2O_2, and slides were blocked using a protein blocking reagent (Dako) at room temperature. Tissue sections were incubated with primary antibodies overnight at 4° C (PRR: Gene Tex, 1:50 000; p-AKT: Cell signaling, 1:50) and then with their respective biotin-conjugated secondary antibodies (Santa-Cruz). Staining was developed using DAB (Sigma-Aldrich) containing 0.015% H_2O_2. Counterstaining was performed with hematoxylin.

Lysotracker image processing

Using a Zeiss microscope with 20X objective and an AxioCam HR camera, four to six images were taken from each well. *Image J* software was used to process the images. Cells on the bright-field images were manually counted using the *Image J* cell counter plugin. The mean gray value of images was then measured using the same software. Mean gray values were added and divided by the total number of cells counted from the corresponding bright-field images to find the mean gray value per cell.

Tumor tissue sample preparation

The dissected tissues were frozen at –20° C with OCT compound (Tissue-Tek; Miles Scientific), and 5-µm slices were cut and immediately fixed in formalin to perform H&E staining for pathological examination. Tumor zones were delimitated together with the adjacent non-cancerous tissues by a clinical pathologist, and further dissections were performed accordingly on the

frozen tissues to isolate tumor regions. To prepare tissue lysates, frozen tissues were washed with nano-pure RNase-free water (Wisent) to remove all apparent traces of OCT compound. The tissues were then finely grinded in liquid nitrogen and lysed in RIPA buffer (50 mM Tris, 150 mM NaCl, 0.1% SDS, 0.5% sodium deoxycholate, 1% Triton-X-100 and 1% NP40) with freshly added protease inhibitors (Complete Mini, Roche). Samples were incubated for 20 minutes on ice and vortexed every 10 minutes before being clarified by centrifugation (17,000 × g, 4° C, 30 minutes). Protein concentrations were determined using the Bicinchoninic Acid assay (Pierce).

TCGA prostate cancer mRNA levels.

To evaluate the clinical relevance between *PRR/ATP6AP2* gene expression and clinical Gleason score, we included the annotated TCGA samples Gleason-6 (n = 49), Gleason-7 (n = 276), Gleason-8 (n = 63), Gleason-9 (n = 129) and Gleason-10 (n = 3) but excluded samples Gleason-N/A (n = 28). Mean Centered *PRR/ATP6AP2* log2 transformed data were filtered based on log2 ratio differences greater than 2. Significance was determined by Wilcoxon rank *T*-test ($P < 0.05$). The R console (version R 3.3.2), Bioconductor source (http://bioconductor.org) and TIGR MultiExperiment Viewer software package MeV version 4.8.1 [1] were used to perform microarray data analysis.

REFERENCE

1. Saeed AI, Bhagabati NK, Braisted JC, Liang W, Sharov V, Howe EA, Li J, Thiagarajan M, White JA, Quackenbush J. TM4 microarray software suite. Methods Enzymol. 2006; 411:134–93. https://doi.org/10.1016/S0076-6879(06)11009-5. [PubMed]

Supplementary Table 1: Clinical parameters of the Centre Hospitalier de l'Université de Montréal TMA patient cohort

**TMA Series**			**TF123**
**Number of patients**			**285**
Age at Diagnosis (years)	Mean		62
	Median		63
	Percentile	25	59
		50	63
		75	67
Follow-up (months)	Mean		128
	Median		129
	Percentile	25	76
		50	129
		75	174
Biochemical Relapse	No		169
	Yes		116
Biochemical Relapse Type	0.2 and Rising		74
	Failed Radical Prostatectomy		42
Bone Metastasis	No		258
	Yes		27
Castrate Resistant	No		258
	Yes		27
Death	PCa specific		19
	Other cause		30
	Overall		49
Gleason Score Radical Prostatectomy	≤3+3		138
	3+4		94
	4+3		19
	≥4+4		29
	Unknown		5
pTNM	2		64
	2a		40
	2b		52
	2c		49
	3a		50
	3b		22
	3c		8
Lymph Node Invasion	No		196
	Yes		9
	Unknown		80
Capsular Penetration	No		205
	Yes		80
Seminal Gland Invasion	No		269
	Yes		16
Margin status	Negative		185
	Positive		95
	Unknown		5

Supplementary Table 2: Correlation values of p-Akt staining with Biochemical Recurrence (BCR), pre-operative PSA levels, and surgical margins status (SMS) on TMA analysis

Clinico-pathological parameter		BCR	Pre-op PSA	SMS
p-Akt	Correlation Coefficient	0.193**	0.144*	0.141*
Spearman's rho	Sig. (2-tailed)	0.001	0.016	0.02
correlation	N	280	280	275

Supplementary Table 3: Clinical parameters of patients from Centre Hospitalier Universitaire de Sherbrooke sampled for normal adjacent and tumor tissues

Patient number	Biobanking (REF)	Gleason Score	PSA at Diagnosis	Prostate weight (g)	Extra-prostatic extension*	Invaded Seminal Vesicles*	pT	pN	pM	Margins **	Biochemical recurrence ***	Metastasis ****
46	–110	3+3	3.6	126	0	0	pT2c	0	x	0	0	0
48	–152	4+3	9	46	1	0	pT3b	x	x	0	1	0
57		3+3	6.6									
72	–149	4+3	7.45	56	1	1	pT3b	0	X	0	1	2
83	–103	3+4	2.9	34	0	0	pT2c	x	x	0	0	0
84	–98	4+3	9.1	84	0	0	pT2c	x	x	0	1	0
86		3+4	15.2									
97	–97	3+4	18.4	54	0	0	pT2b	0	x	2	0	0
105	–96	3+3	9.7	116	0	0	pT2b	x	x	1	0	0
109	–95	4+3	5.7	66	0	0	pT2c	x	x	0	0	0
113	–143	3+3	5.6	42	0	0	pT2b	x	x	0	1	0
120	–90	3+3	7.2	36	0	0	PT2a	x	x	1	0	0
136	–137	3+4	12	40	N/A	0	pT2a	0	x	1	1	0
137	–79	4+3	4.5	28	1	0	pT3	x	x	0	0	0
154	–73	4+3	6.7	42	1	0	pT3a	x	x	2	0	0
158	–129	3+4	36.5	75	1	0	pT3a	1	x	0	1	0
179	–55	3+3	4.4	50	0	0	pT2c	x	x	0	0	0
180	–54	4+3	5.32	40	1	1	pT3b	x	x	2	0	0
194	–123	3+4	3.46	34	0	0	pT2c	x	x	0	1	0
195	–122	3+4	9.6	102	0	0	pT2c	x	x	1	1	0

*0 = No, 1 = Yes, **0: Negative, 1: Unifocal, 2: Multifocal, ***1: yes if > 0.2 ng/mL, 0: no, ****0: None, 1: Lymph node, 2: bones, 3: visceral.

Supplementary Table 4: Clinical parameters of patients from the Centre Hospitalier Universitaire de Sherbrooke sampled for plasma and urine ELISA

**Plasma specimens**		
**Number of patients**		**155**
Age at Diagnosis (years)	Mean	62
	Median	62
	Percentile 25	58
	Percentile 50	62
	Percentile 75	66
Follow-up (Months)	Mean	117
	Median	124
	Percentile 25	65
	Percentile 50	124
	Percentile 75	168
Biochemical Relapse	No	106
	Yes	49
Biochemical Relapse Type	0.2 and Rising	29
	Failed Radical Prostatectomy	20
Bone Metastasis	No	137
	Yes	18
Castrate Resistant	No	138
	Yes	17
Death	Prostate cancer specific	13
	Other cause	12
	Overall	25
Gleason Score Radical Prostatectomy	≤3+3	78
	3+4	50
	4+3	11
	≥4+4	13
	Unknown	3
pTNM	2	44
	2a	18
	2b	34
	2c	14
	3a	26
	3b	15
	3c	4
Lymph Node Invasion	No	106
	Yes	5
	Unknown	44
Capsular Penetration	No	110
	Yes	45
Seminal Gland Invasion	No	146
	Yes	9
Margin status	Negative	99
	Positive	53
	Unknown	3

Supplementary Table 5: RT-PCR primer sequences

Gene	Primer name	Sequence (5′–3′)
Insulin-likegrowthfactorbinding protein 2	*IGFBP2* Fwd	GGAGGAGCCCAAGAAGCTGCG
Insulin-like growth factor binding protein 2	*IGFBP2* Rev	CCCGTTCAGAGACATCTTGCACTG
ATPase, H+ transporting, lysosomal accessory protein 2	*HT028* Fwd	GACACCTCCCTCATTAGGAAGAC
ATPase, H+ transporting, lysosomal accessory protein 2	*HT028* Rev	GATCATTATCCAAAGTACCATGTTG
Calsyntenin 1 (CLSTN1)	*CLSTN2* Fwd	GGAACACCGCTCCTTTGTTGACCTG
Calsyntenin 1 (CLSTN1)	*CLSTN2* Rev	CCGCATGGTCCGCCGATGTG
Vinculin	*VCL2* Fwd	CTACTAGGCAAAATGAGAGGGCAG
Vinculin	*VCL2* Rev	GAAGGAAGGATGTTCAGGCAAGG
Phosphatase and Tensin homolog	*PTENa* FWD	CAAGTTCATGTACTTTGAGTTCCC
Phosphatase and Tensin homolog	*PTENa* REV	GCACGCTCTATACTGCAAATGC
Protein tyrosine phosphatase, receptor type, F	*LAR* Fwd	ACGCTCTGCCAACTACACC
Protein tyrosine phosphatase, receptor type, F	*LAR* Rev	CACAAGATCAATCGGAGGCTT
Desmoglein 2	*DSG2* Fwd	TGATGCTGCTGCTTAGGTGCC
Desmoglein 2	*DSG2* Rev	CCTGGGTTGCTTTACTGGACGGC
Chaperonin containing TCP1, subunit 8 (theta)	*CCT8* Fwd	AGGAGGGAGCGAAACACTTTT
Chaperonin containing TCP1, subunit 8 (theta)	*CCT8* Rev	GCAGGATGCTGTACTTCTAGTTC
Ribonuclease/Angiogenin Inhibitor 1	*RNH1* Fwd	CCTGCTGCTGCTCTCCCTGG
Ribonuclease/Angiogenin Inhibitor 1	*RNH1* Rev	GGCAGAAATAAGCGGATCTGAGCGT
Spondin 2	*SPO2* Fwd	GAGGGCACAGGGGGTTTCGC
Spondin 2	*SPO2* Rev	AGAGCAGACGGGACACGGGG
Prostate specific antigen precursor	*PSA* Fwd	TGAACCATGTGCCCTGCCCG
Prostate specific antigen precursor	*PSA* Rev	AGGGGTGCTCAGGGGTTGGC
Cystatin C	*CST3* Fwd	TGCGGCGTGCACTGGACTTT
Cystatin C	*CST3* Rev	GCTACGATCTGCTTGCGGGCG
Alpha-1 collagen VI	*COL6A1* Fwd	ACACCGACTGCGCTATCAAG
Alpha-1 collagen VI	*COL6A1* Rev	CACCGAGAAGACTTTGACGC
Alpha actinin 4	*ACTN4* Fwd	TGGAGGTCATATCAGGGGAGC
Alpha actinin 4	*ACTN4* Rev	CTTCTGCCCCGATGGAGAC
Glyceraldehyde-3-phosphate dehydrogenase	*GAPDH* Fwd	AGGGCTGCTTTTAACTCTGGT
Glyceraldehyde-3-phosphate dehydrogenase	*GAPDH* Rev	CCCCACTTGATTTTGGAGGGA

Supplementary Table 6: PRR shRNA and siRNA sequences

PRR shRNA/siRNA	Name	Sequence (5′–3′)
shRNA 1	TRCN0000038605	CCGGGCAGTGTCATTTCGTACCCTTCTCGAGAAGGGTACGA AATGACACTGCTTTTTG
shRNA 2	TRCN0000308100	CCGGAGTCTTGACAGTGTTGCAAATCTCGAGATTTGCAACA CTGTCAAGACTTTTTG
shRNA 3	TRCN0000289297	CCGGCCTGGATATGATAGCATCATTCTCGAGAATGATGCTATC ATATCCAGGTTTTTG
siRNA 1	SASI_Hs01_00147063	GAGUGUAUAUGGUAGGGAA
siRNA 2	SASI_Hs01_00147062	GUCUUGACAGUGUUGCAAA

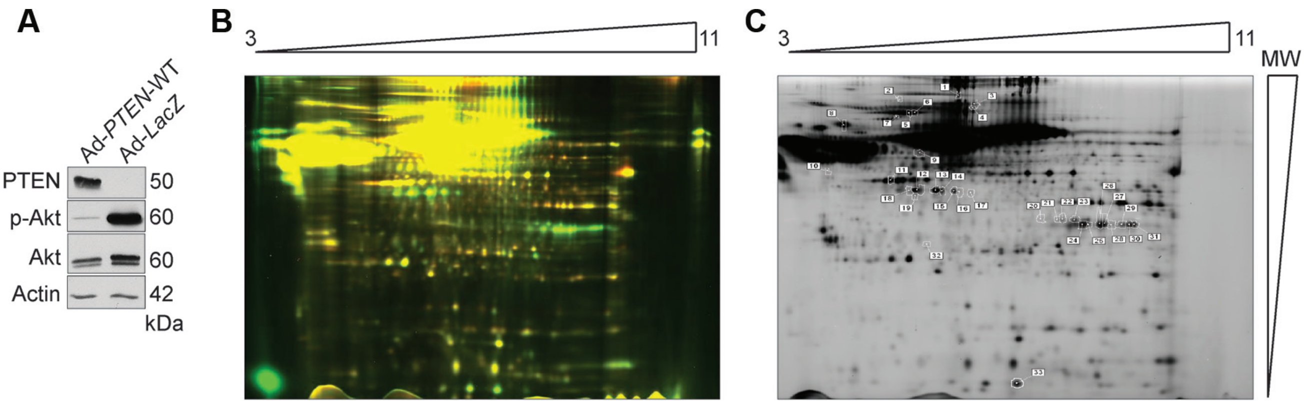

Supplementary Figure 1: Identification of secreted factors regulated by PTEN. (**A**) Western blot analysis demonstrating PTEN expression and p-Akt downregulation in the presence of PTEN in whole cell extracts of Ad-PTEN WT infected LNCaP cells compared to Ad-LacZ infected controls. Total Akt was used as a control for p-Akt levels and Actin was used as a loading control. (**B**) Representative scan of a 2D-DiGE gel where proteins isolated from conditioned media of LNCaP cells infected with wild type PTEN Adenovirus (Ad-PTEN WT) were labelled with Cy5 dye (red), and proteins from cells infected with LacZ Adenovirus (Ad-LacZ) labelled with Cy3 dye (green). (**C**) Representative silver stained gel labelled for spots identified by mass spectrometry.

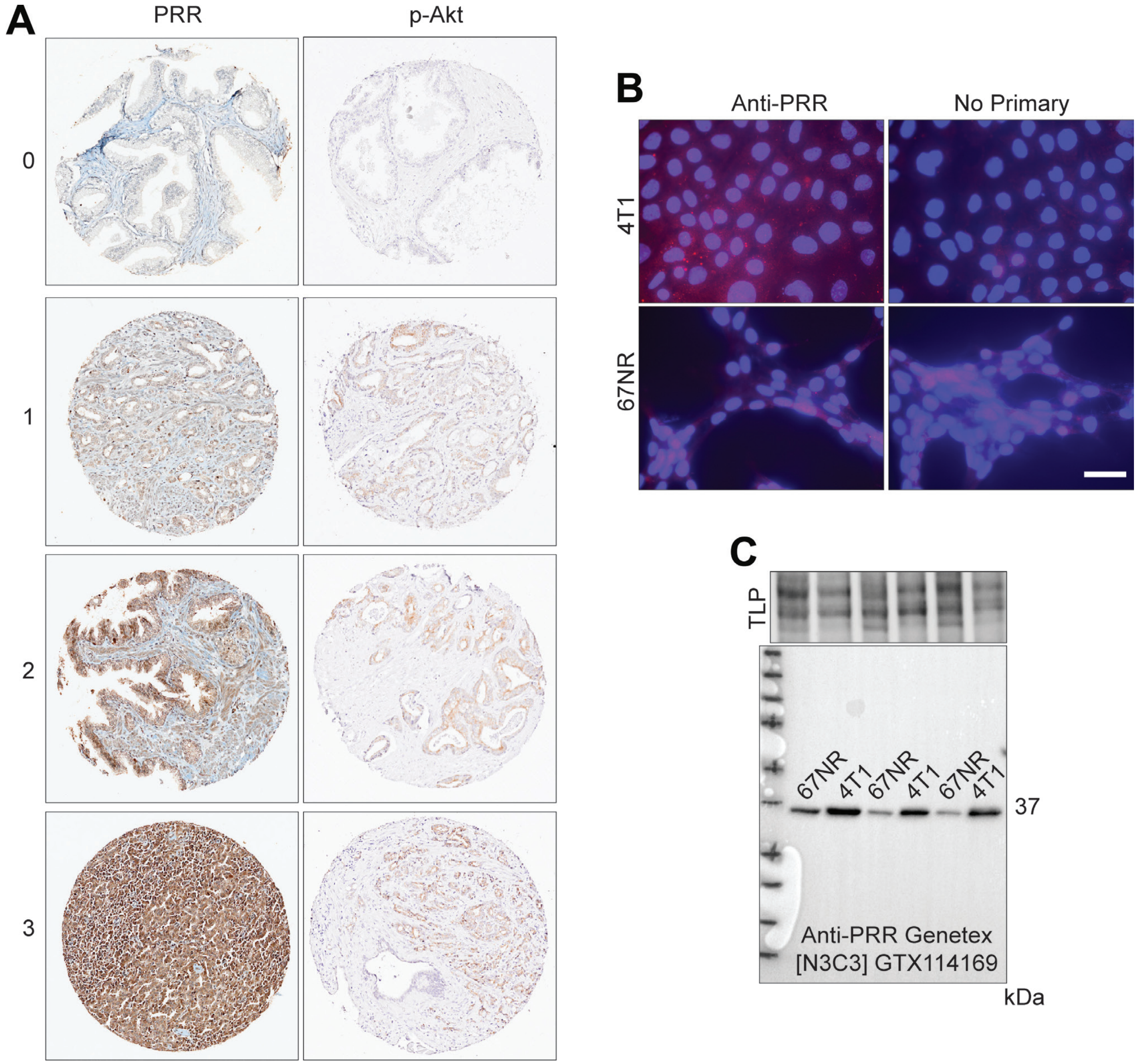

Supplementary Figure 2: PRR and p-Akt TMA scoring and PRR antibody TMA validation. (**A**) Representative scoring of immunohistochemistry staining intensities for p-Akt and PRR in prostate cancer TMAs. (**B**) Immunofluorescence images (20×) of murine breast cancer cells 4T1 (High PRR expression) and 67NR (Low PRR expression) stained with the anti-PRR antibody used in TMA staining (Genetex [N3C3] GTX114169) or No primary antibody for control. Scale bar measures 50 µm. (**C**) Western blot analysis demonstrating the band level of PRR expression and the specificity of the antibody used to detect PRR in 3 replicates of 67NR and 4T1 cell lines. Total lane protein was used as a loading control.

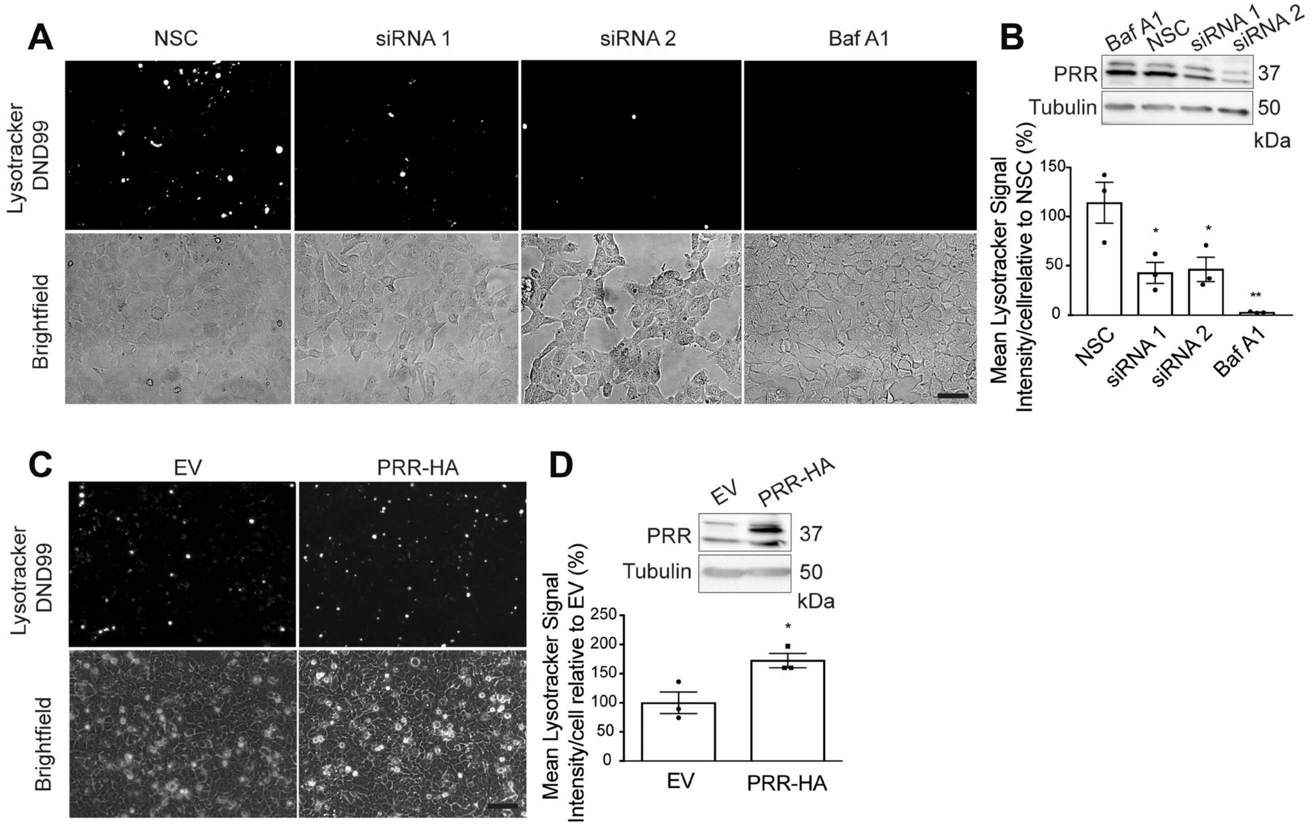

Supplementary Figure 3: PRR expression affects cell growth and V-ATPase activity in HEK 293T cells. (**A**) LysoTracker DND-99 and corresponding brightfield images (20X) of Non-Silencing siRNA (NSC) (50 nM) transfected, PRR siRNA 1 or siRNA 2 (50 nM) transfected, or Bafilomycin A1 (Baf A1) (100 nM) treated HEK-293T cells. (**B**) Quantification of Mean LysoTracker signal per cell shows a decrease in LysoTracker signal after knocking down PRR using siRNA 1 ($^{*}P = 0.036$), siRNA 2 ($^{*}P = 0.049$), or after Bafilomycin A1 (100 nM) ($^{**}P = 0.0028$) treatment in HEK-293T cells ($n = 4$). Western blot analysis validating PRR knockdown in siRNA1 and siRNA 2 transfected cells. Tubulin was used as a loading control (Top Panel). (**C**) LysoTracker DND-99 and corresponding brightfield images (20X) of empty vector (EV) and PRR-HA transfected HEK 293T cells. (**D**) Corresponding Quantification of Mean LysoTracker signal per cell demonstrates an increase in LysoTracker signal in PRR-HA transfected ($^{*}P = 0.031$) HEK-293T cells ($n = 3$). Western blot analysis validating PRR overexpression in HEK293T cells following PRR-HA transfection. Tubulin was used as a loading control (Top Panel). Scale bar measures 100 μm. Data are presented as the mean ± SEM. Statistical tests conducted using Student's t test.
